# Analytical and computational results regarding scalar property in a striatal-beat frequency model of interval timing

**DOI:** 10.1186/1471-2202-12-S1-P23

**Published:** 2011-07-18

**Authors:** Sorinel A Oprisan, Catalin V Buhusi

**Affiliations:** 1 Department of Physics and Astronomy, College of Charleston, Charleston, SC 29424, USA; 2 Neuroscience Department, Medical University of South Carolina, Charleston, SC 29425, USA

## 

The capability of performing interval timing is essential for survival, adaptation and its impairment leads to sever cognitive and/or motor dysfunctions. Although the localization of brain regions essential for interval timing is not yet clear, some progress has been made. For example, both temporal production and temporal perception are strongly connected to striatum and its afferent projections from the substantia nigra pars compacta [1]. Since interval timing deficiencies were found both in peak-interval procedure [1] and the duration bisection procedure [2], it suggests that lesions of these cortical areas and not simple motor deficiencies are the leading causes. We used a distributed network model - the beat frequency model – for modeling the neural network involved in interval timing. The striatal-beat frequency (SBF) model assumes that neural oscillators, loosely localized in prefrontal cortex, produce beats that have periods spanning a much wider range of periods than their intrinsic values [3]. It is assumed that at the beginning of each trial all oscillators are reset and start in phase. At the time of reinforcement or feedback, the oscillators can be read out to determine whether they are spiking. The small group of neurons that are spiking at the time of reinforcement could be considered to represent a neural code for that duration and could be encoded via Hebbian strengthening of the activated synapses. A temporal prediction could be brought about on a new trial by a threshold driven comparison between the number of strengthened neurons currently firing and the number of neurons that fired previously at the time of reinforcement. We investigated both analytically and numerically the properties of the output function generated by the SBF model. This output function mimics behavioral responses of animals in interval timing experiments. We found analytically and checked numerically that in the absence of any kind of variability in the parameters of the SBF model, the width of the output function that measures the spread of behavioral responses only depends on the number of oscillators and the range of frequencies they cover. Therefore, in the absence of parameter variability, the scalar property is violated. However, if variability is allowed, for example in the memorization/retrieval of criterion time, then the output function of SBF model is always Gaussian, which is a consequence of the central limit theorem, regardless the probability distribution function (pdf) of fluctuating parameter. Moreover, we found that the scalar property is also preserved regardless the pdf of fluctuating parameters.
